# My Tongue is Trying to Kill Me: From Cancer to Psychotic Disorder

**DOI:** 10.1192/j.eurpsy.2026.12136

**Published:** 2026-07-31

**Authors:** D. Polić, E. Žderić, G. Rubeša

**Affiliations:** 1 Department of psychiatry, Teaching Institute of Public Health of Primorsko-Goranska County; 2 Department of psychiatry, University Hospital Centre Rijeka, Rijeka, Croatia

## Abstract

**Introduction:**

Psychiatric complications following oncological procedures are rarely psychotic in nature, especially in individuals without a prior psychiatric history. However, when they occur, they often reflect deep psychological conflicts and symbolic interpretations of bodily trauma.

**Objectives:**

To present a case of delayed-onset psychotic decompensation following surgical treatment for tongue cancer, in a high-functioning individual with no previous psychiatric treatment.

**Methods:**

We describe the clinical trajectory of a 65-year-old male, a successful lawyer with no psychiatric history and a long-standing untreated alcohol use disorder. The patient underwent a wedge excision of a lesion on the left lateral border of the tongue. Histopathological diagnosis confirmed *Carcinoma planocellulare invasivum linguae* (invasive squamous cell carcinoma of the tongue). Persistent oral pain led to prolonged use and abuse of non-opioid analgesics. Three years after surgery, he presented with a psychotic episode characterized by paranoid ideation, ideas of reference, and somatic delusions focused on the tongue and speech. Clinical data were collected through medical record review, psychiatric interviews, and diagnostic evaluation.

**Results:**

The psychotic content was thematically tied to the somatic site — the tongue — previously affected by cancer and surgery. The patient became preoccupied with the sensation and appearance of his oral cavity, expressing bizarre beliefs about its function and others’ reactions to it. Persistent oral pain, despite lack of somatic findings, contributed to his fixation, and was managed with chronic non-opioid analgesic use, complicated by documented abuse of these medications. His alcohol use remained significant but had never led to psychiatric intervention. The onset of psychosis appeared to be a symbolic processing of somatic trauma, complicated by substance use and chronic pain.

**Conclusions:**

This case highlights the need for long-term psychosocial monitoring in patients undergoing oncological treatment, even in the absence of prior psychiatric diagnoses. Psychotic disorders may emerge as delayed responses to deeply personal somatic trauma, particularly when compounded by addiction and chronic pain managed with long-term and abused non-opioid analgesics. An integrated, multidisciplinary approach is essential for early recognition and intervention.

**Disclosure of Interest:**

None Declared

